# Prevalence and determinants of anaemia among men in rural India: Evidence from a nationally representative survey

**DOI:** 10.1371/journal.pgph.0001159

**Published:** 2022-12-01

**Authors:** Aditya Singh, Sumit Ram, Shivani Singh, Pooja Tripathi

**Affiliations:** 1 Department of Geography, Banaras Hindu University, Varanasi, Uttar Pradesh, India; 2 India Health Action Trust, Lucknow, Uttar Pradesh, India; University of Minnesota, UNITED STATES

## Abstract

Anaemia among men is a significant health issue which has not been given due importance. Only a handful of studies have captured the prevalence of anaemia among men. There is dearth of evidence base on anaemia among men in India. Therefore, this study attempts to fill this research gap by examining the socioeconomic, geographic, health-related, and behavioural differentials of anaemia among rural men in India. We analysed a cross-sectional sample of 61,481 men aged between 15–54 and living in rural areas from the National Family Health Survey (NFHS-5), conducted in 2019–21. Bivariate statistics and multivariable logistic regression were employed to assess the factors associated with anaemia. In rural India, three out of ten men were found to be anaemic. Older men [49–54 years] (Odds Ratio: 1.10, 95% CI, 1.00–1.21), men without a formal education (OR: 1.36, 95% CI, 1.26–1.47), those from Scheduled Tribes (OR: 1.48, 95% CI, 1.39–1.58) and men who belonged to the poorest wealth quintile (OR: 1.24, 95% CI: 1.25–1.35) had a higher risk of anaemia. Men who were underweight were more likely to be anaemic (OR: 1.36, 95% CI: 1.30–1.43). When compared to the central region, men from the eastern (OR: 1.47, 95% CI: 1.39–1.55) parts of India had higher a risk of anaemia. The findings suggest the need to recognise anaemia among men as a public health issue. When developing policy, significant variation in socioeconomic, geographic, health-related, and behavioural factors must be taken into account. Men should also be screened on a regular basis in order to reduce the national burden of anaemia.

## Introduction

According to the World Health Organization (WHO), anaemia is a disorder in which the number or haemoglobin concentration of red blood cells is below normal which subsequently results in the decreased oxygen-carrying capacity of blood [1] Haemoglobin is an iron-containing protein in the red blood cells (RBC) that transports oxygen from the lungs to the tissues and carries carbon dioxide from tissues back to lungs [2]. Nutritional deficiencies, particularly iron deficiency is the main reason behind this disease although deficiencies in vitamins B9, B12 and A may also cause anaemia. Acute and chronic infections, and genetic haemoglobin disorders are also found to lead to anaemia [3]. Although the degree to which anaemia in a population can be attributed to these causes varies across populations [4].

Despite great breakthroughs in science and healthcare, anaemia continues to be a significant global public health issue. Every fourth person in the world (27%) has anaemia, with the developing countries alone accounting for more than 89% of the burden [4]. Although anaemia is a condition that affects all age groups, it is more common among pregnant women and children. Therefore, there have been global efforts to reduce anaemia among adolescent girls and boys, and women of reproductive age (WRA), especially pregnant and lactating women. Reducing anaemia among WRA by 50% is one of the prime goals in six global nutrition targets for 2025 endorsed by WHO [5] and putting an end to all forms of malnutrition has been listed as one of the targets of the Sustainable Development Goal (SDGs) 2 [6].

Developing countries like India have also made continuous efforts to reduce the anaemia among children and women. However, as suggested by the Global Nutrition Report (2021), there has been little progress in combatting anaemia and malnutrition since 2016 [7]. It is also evident in India’s rank (94th among 107 countries) in the recently published Global Hunger Index. Moreover, none of the programs and interventions have addressed anaemia among men, despite the fact that one in every four men in India suffer from anaemia [8].

While anaemia during pregnancy and early childhood is linked to a variety of negative outcomes for the child, including low birth weight, delayed mental and cognitive development, mortality and has the potential to cause maternal mortality [9–12], anaemia in men is generally not considered a disease or a significant problem. Though anaemia among men is rarely fatal, still it can cause fatigue, difficulty concentrating, and lethargy, which not only reduces quality of life but is also thought to decrease economic productivity [13,14]. As per the India State-Level Disease Burden Initiative, iron-deficiency anaemia is one of the major causes resulting to burden of morbidity among men across all states and union territories (UTs) of India [15]. Therefore, anaemia among men should also be treated as a serious public health issue.

While there is an abundant literature from developing and underdeveloped countries including India that focuses on anaemia among WRA and their children, [16–18], there is dearth of evidence base on anaemia among men, especially in India [19]. The literature is much more limited particularly for rural men.

A study in 2019 focused on the differentials of anaemia prevalence among men in India [19]. A recent study by Kumar et al. (2021) attempted to decompose the factors that contribute to socioeconomic inequality in anaemia among men in India [20]. Also, Kumar et al. assessed the correlates and geographic distribution of anaemia in men in the empowered action group (EAG) states of India [21]. However, no study has ever focused on the prevalence of anaemia among men in rural India or the risk factors associated with it.

Moreover, anaemia among men is on the rise, according to the most recent National Family Health Survey, 2019–21 (NFHS-5), and is more prevalent in rural areas than in urban areas [8]. Because the characteristics and health behaviours of rural males differ significantly from those of urban men, it is crucial that they be researched separately from urban men. In order to be able to design and implement targeted interventions to reduce anaemia among rural men, it is crucial to identify not only the geographical regions where anaemia prevalence is high but also the vulnerable groups of rural men who are more likely to suffer from anaemia. Therefore, using data from the nationally representative survey, this study aims to examine socioeconomic, health-related and behavioural differentials in the prevalence of anaemia among rural men in India and the factors associated with it. The spatial distribution of anaemia among rural men across Indian states and districts is also discussed in this study.

## Data and methods

### Data source

The data comes from the latest round of National Family Health Survey (NFHS-5) carried out by International Institute for Population Sciences during 2019–2021 under the supervision of Ministry of Health & Family Welfare, Government of India. NFHS, the Indian version of the Demographic and Health Surveys (DHS), is a nationally representative cross-sectional survey that collects data on a wide range of demographic, socioeconomic, and health-related issues.

Using a two-stage stratified random sampling method, NFHS-5 interviewed 724115 women aged 15 to 49 years and 101839 men aged 15–54 years from 636699 households. Response rate was 97% and 92% for women and men respectively. Through a series of biomarker tests and measurements, the clinical, anthropometric, and biochemical (CAB) component of the NFHS-5 provided critical estimates of the prevalence of malnutrition, anaemia, hypertension, high blood glucose levels, waist and hip circumference, Vitamin D3, HbA1c, and malaria parasites. The survey covered 707 districts from 28 states and 8 UTs. A uniform sample design, which is representative at the national, state/UT, and district level, was adopted in each round of the survey [8].

The Biomarker Schedule contained measurements of height, weight, and haemoglobin levels for children; measurements of height, weight, waist and hip circumference, and haemoglobin levels for women aged 15–49 years and men aged 15–54 years; and blood pressure and random blood glucose levels for women and men aged 15 years and over. Additionally, both men and women were requested to provide a few more drops of blood from a finger prick for laboratory testing for HbA1c, malaria parasites, and Vitamin D3 [8].

We made a request to the DHS Program to provide us with the NFHS data. Once we received the permission to use the data, we downloaded the Men’s data file (MR) and the Household Member data file (PR). MR and PR datasets were then merged to avail the information on anaemia among men in India.

### Ethics approval and consent to participate

The present study has used secondary data, which is available in the public domain. The dataset has no identifiable information of the survey participants. Therefore, no ethical approval is required for this study.

### Sample

**Fig 1:**
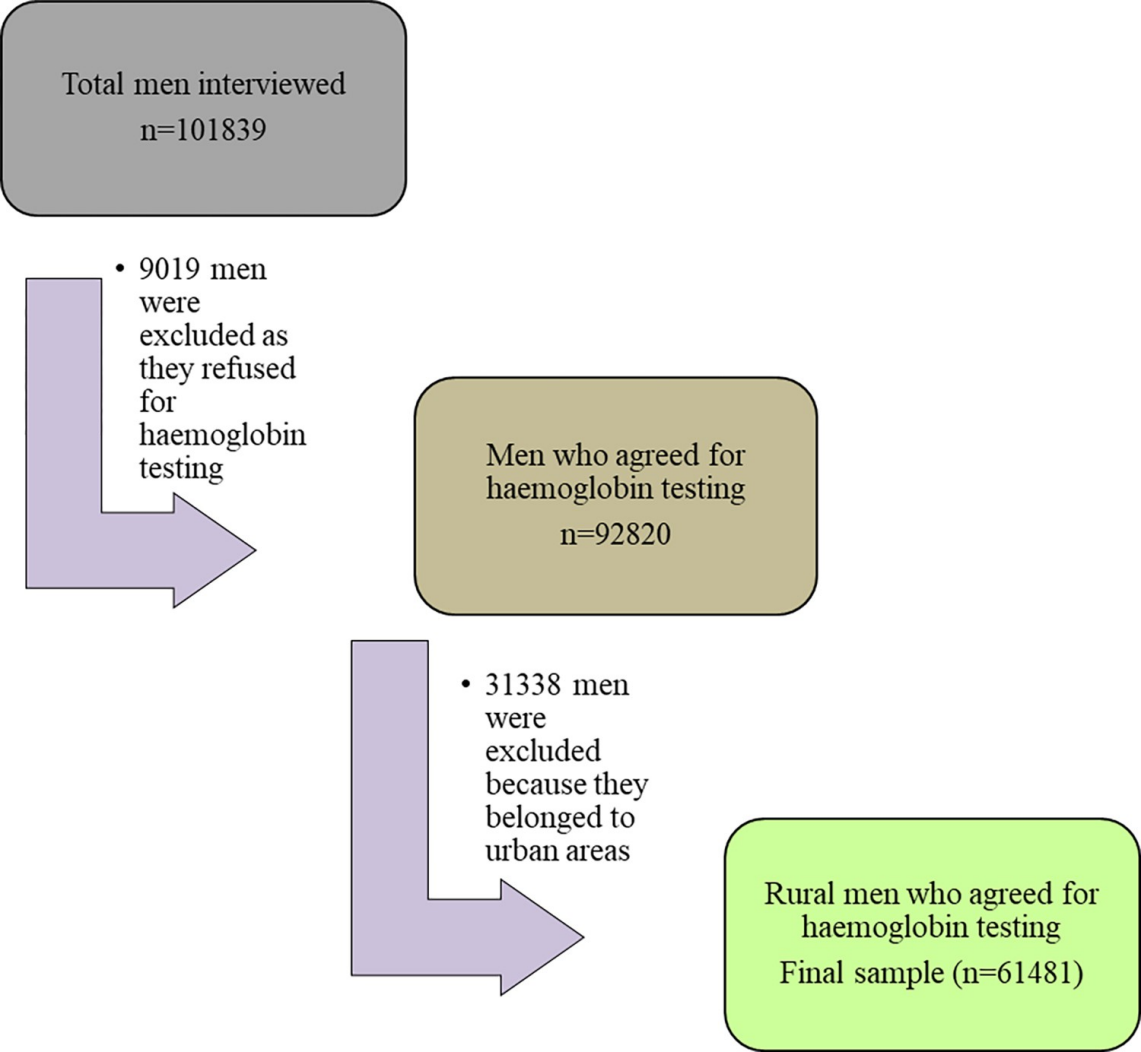
Sample selection for the present study.

Fig 1 depicts the process of sample selection for the present study. Out of the 111,179 eligible men aged 15–54 years selected for the state module, 101839 men who were normally inhabitants and spent the night before the survey in their homes were interviewed. 92820 men consented to have their haemoglobin levels checked. For this study, 31339 out of the 92820 men were excluded as they belonged to urban areas. Our study was limited to remaining 61481 men residing in rural areas.

### Anaemia testing

The authors did not collect blood specimens for anaemia testing for this study. These were collected under NFHS-5 by health investigators from eligible men aged 15 to 54 with their consent. Blood samples were drawn from a drop of blood taken from a finger prick (or a heel prick for children age 6–11 months) [8] and collected in a microcuvette, a single-use pipette. Concentration of haemoglobin was analysed on-site with HemoCue Hb 201+ analyser. Introduced in 1990, the HemoCue Hb 201+ is a battery-operated portable device used for quantitative determination of haemoglobin level in undiluted, capillary or venous blood. It converts the haemoglobin into methemoglobin and combines it with azide to form azidemethemoglobin followed by measurement of transmittance and haemoglobin absorbance [22–24].

### Dependent variable

The dependent variable in this study was whether or not the respondents were anaemic. Men were considered to have anaemia in any form if their haemoglobin concentration was less than 13.0 g/dL, mildly anaemic if it was 12.0–12.9 g/dL, moderately anaemic if it was 9.0–11.9 g/dL, and severely anaemic if it was less than 8.9 g/dL, according to WHO criteria [25]. The study developed a dichotomous variable for prevalence of anaemia. Men with a haemoglobin level less than 13g/dl were considered ‘anaemic’ and coded ‘1’ while men having a haemoglobin level greater than 13g/dl were classified as ‘not anaemic’ and coded ‘0’. We did not take into account the three categories of anaemia: mild, moderate, and severe.

### Independent variables

**Fig 2:**
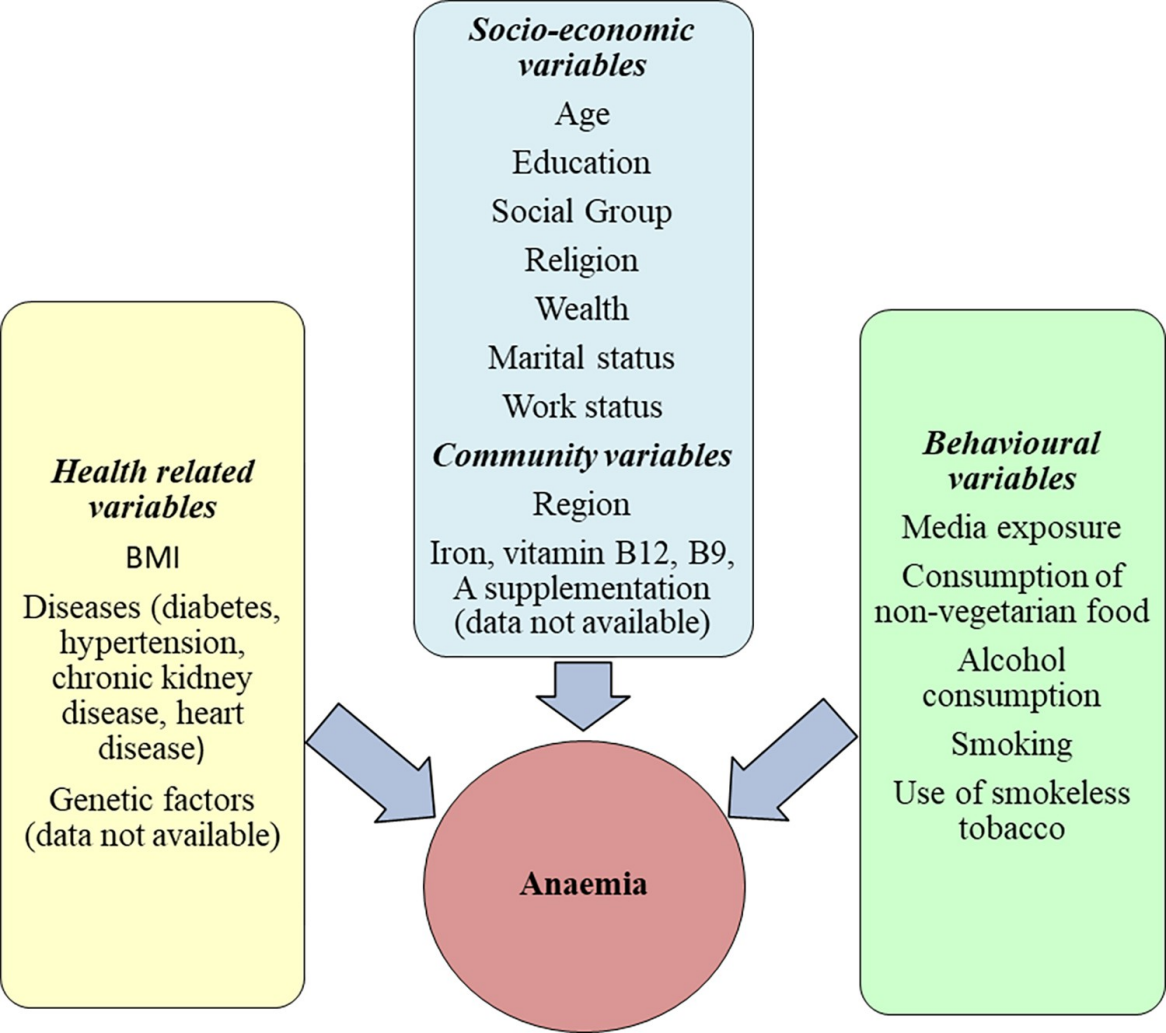
Conceptual framework showing factors affecting anaemia.

Definitions, categories and codes of independent variables are enlisted in Table 1. A wide range of variables were found to predict anaemia among men [26–28]. To illustrate, as a proxy for household income, the wealth index was chosen as a gauge of economic inequality. This is a measure of household wealth that is determined to be reliable based on both expenditure and income metrics [8]. Wealth index was one of the key predictors of this study. BMI was also one of the significant determinants of anaemia among men which was classified into four categories i.e., underweight (<18.5 kg/m^2^), normal (18.5–24.99 kg/m^2^), overweight (25–32 kg/m^2^), and obese (>32kg/m^2^). Age, education, social group, religion, and alcohol consumption were other important factors of anaemia in men. These variables could be categorised into four domains namely socioeconomic factors, community variables, health-related variables and behavioural characteristics. All the variables in present study were selected only after extensive review of literature and according to data availability. Fig 2 depicts a conceptual framework that shows the factors affecting anaemia among men.

**Table 1:** Description of the variables.

Independent variables	Description (Codes)
***Socio-economic variables***	
Age (in years)	Age of men has five categories: 15–19 (0), 20–29 (1), 30–29 (2), 40–49 (3), 49–54 (4)
Level of education	Highest education level achieved by man is classified into four categories: no education (0), primary- 1–5 years (1), secondary- 6–10 years (2), higher- 11 and above (3)
Social group	Social group has four categories: Others (0), Scheduled Caste (1), Scheduled Tribe (2), Other Backward Classes (3)
Religion	Religion has four categories: Hindu (1), Muslim (1), Christian (2) and others. Other included all religious groups other than Hindu, Muslim and Christian
Household wealth	Household wealth is divided into five categories: poorest, poorer, middle, richer and richest. For the calculation of wealth index, households were given scores based on the number and kinds of consumer goods they own, ranging from a television to a bicycle or car, and housing characteristics such as source of drinking water, toilet facilities, and flooring materials. These scores were derived using principal component analysis. National wealth quintiles were compiled by assigning the household score to each usual (de jure) household member, ranking each person in the household population by their score, and then dividing the distribution into five equal categories, each with 20% of the population [8].
Work status	Whether a man currently works or not: no (0) and yes (1)
Marital status	Marital status has three categories: never married (0), currently married (1), and formerly in union (2) which includes widowed, separated, divorced and deserted.
***Geographic variable***	
Region	A region in this study is a group of Indian states and union territories: Central region (0) includes the states of Uttar Pradesh, Madhya Pradesh and Chhattisgarh; South region (1) includes the states of Kerala, Karnataka, Andhra Pradesh, Tamil Nadu, Telangana and the UTs of Andaman & Nicobar Islands, Pondicherry and Lakshadweep; North region (2) includes Ladakh, Jammu & Kashmir, Himachal Pradesh, Uttarakhand, Punjab, Rajasthan, Haryana, Chandigarh, and Delhi; East region (3) includes the states of Bihar, Jharkhand, West Bengal, and Odisha; West region (4) includes the states of Gujarat, Maharashtra, Goa, and Dadra & Nagar Haveli and Daman & Diu; North-East region (5) includes the states of Sikkim, Assam, Meghalaya, Manipur, Mizoram, Nagaland, Tripura, and Arunachal Pradesh [8]
***Health related variables***	
Body Mass Index	It is defined as the ratio between the weight of a man in kilogram divided by the squared height in meter. Body mass index is divided into four categories underweight (<18.5 kg/m^2^), normal (18.5–24.99 kg/m^2^), overweight (25–32 kg/m^2^) and obese (> 32 kg/m^2^)
Blood sugar level	It has three categories: normal (0), random blood glucose less than 140 g/dl; high (1), random blood glucose level of 141–160 mg/dl; and very high (2), random blood glucose level of more than 160 mg/dl.
***Behavioural variables***	
Media exposure	It refers to how often men watch television, read newspapers, and listen to the radio. Exposure to mass media was defined as men using them every day or at least once a week [coded as yes (1)]. Men who used it less than once a week or never [marked as no (0)] were regarded to have no media exposure.
Consumption of non-vegetarian food	Men’s consumption of egg, fish, and chicken/meat. Men who consumed the above-mentioned foods every day and at least once a week were classified as eating non-vegetarian food [coded as yes (1)], and men who ate them less than once a week or not at all were classified as not eating non-vegetarian food [coded as no (0)]
Alcohol consumption	Frequency of drinking alcohol having four categories: never (0), less than once in a week (1), once a week (2), daily (3)
Currently smokes	Men using any of bidi, cigarette, hookah or cigar were coded as ‘1’ yes and 0 ‘no’ otherwise.
Use of smokeless tobacco	Men using forms of smokeless tobacco (gutkha with paan, khaini, nassi) were coded as ‘1’ yes and 0 ‘no’ otherwise.

### Statistical analysis

Bivariate statistics was used to analyse the prevalence of anaemia among rural men by their background characteristics. The analysis was weighted for two-stage sampling design. Thus, weighted estimates were presented. Sampling weights (importance weight: iw) were included in the study. The ‘svyset’ command was used in Stata to account for clustering at the PSU level.

Since our dependent variable was binary in nature, we employed binary logistic regression to assess the effects of the predictor variables on the dichotomous dependent variable of the study. Chi-square test was performed to check if the independent variables were associated with the dependent variable. Only those variables that were found statistically significant (p<0.05) were included in the regression models. We applied four models, i.e., model 1 included socioeconomic variables; model 2 included statistically significant variables from model 1 variables and geographic variable. Model 3 contained significant variables from model 2 and health-related variables. The final model included significant variables from model 3 and behavioural variables. The equation of a single-level binary logistic regression model can be specified as:

$$Log\;(P/1‐P)=\beta_{0}+\beta_{1}x_{1}{+}_{\ldots }+\beta_{2}x_{2}$$

Where, P indicates the probability of an event (prevalence of anaemia in this study), β_0_ is the intercept on y axis, βi indicates the regression coefficients associated with the reference group, x_i_ is the independent variable.

The results of logistic regression models are presented in the form of odds ratios with p-values and 95% confidence intervals (CI). We calculated Variance Inflation Factors (VIFs) for the final model to check whether multicollinearity among the independent variables existed. The VIFs for all the independent variables were considerably small (below 2.5) indicating that multicollinearity was not a problem for the models (results not shown). Stata 16 was used for analysing the unit level data [29]. ArcMap (version 10.5) was used to create the choropleth maps [30].

## Results

### Profile of the respondents

Table 2 present the sociodemographic profile of the men in rural India. About 17% men were aged between 15–19 years. One in every seven men had no formal education. About a quarter of all men belonged to SC group, while 12.4% men belonged to ST group. The Hindu faith was practised by the vast majority of men (81%). Nearly one-fourth men belonged to the poorest wealth quintile. Around 30% men were from the eastern region of India. At the time of the survey, roughly two-thirds of men were married. About 18% of men were underweight (BMI less than 18.5 kg/m2). Around 28% of men used smokeless tobacco, while 45% smoked cigarettes.

**Table 2:** Profile of the respondents, India, 2019–21.

Background Characteristics	n	%
**Age (in years)**		
15–19	10329	16.8
20–29	17061	27.8
30–39	15592	25.4
40–49	13157	21.4
50–54	5349	8.7
**Level of education**		
No education	8853	14.4
Primary	8423	13.7
Secondary	35597	57.9
Higher	8607	14.0
**Social group**		
SC	14202	23.1
ST	7624	12.4
OBC	27543	44.8
Others	12173	19.8
**Religion**		
Hindu	8546	81.0
Muslim	8546	13.9
Christian	1721	2.8
Others	1414	2.3
**Household wealth**		
Poorest	14878	24.2
Poorer	16047	26.1
Middle	14694	23.9
Richer	11067	18.0
Richest	4734	7.7
**Marital status**		
Never married	22555	34.2
Currently married	42465	64.3
**Work status**		
No	14817	24.1
Yes	46664	75.9
**Region**		
South	14264	23.2
North	5164	8.4
Central	7808	12.7
East	17891	29.1
West	12235	19.9
North-East	4119	6.7
**Body Mass Index**		
Underweight	11067	18.0
Normal	38057	61.9
Overweight	10390	16.9
Obese	1967	3.2
**Media exposure**		
No	24285	39.5
Yes	37196	60.5
**Consumption non-vegetarian food**		
No	19182	31.2
Yes	42299	68.8
**Alcohol consumption**		
Never	47340	77.0
Less than once a week	5841	9.5
Once a week	6087	9.9
Almost every day	2213	3.6
**Currently smokes**		
No	33876	55.1
Yes	27605	44.9
**Use of smokeless tobacco**		
No	44020	71.6
Yes	17461	28.4
**Total**	61481	100.00

**Notes:** n = Sample of rural men who agreed for haemoglobin testing, All % are weighted.

### Differentials in prevalence of anaemia by background characteristics

**Fig 3:**
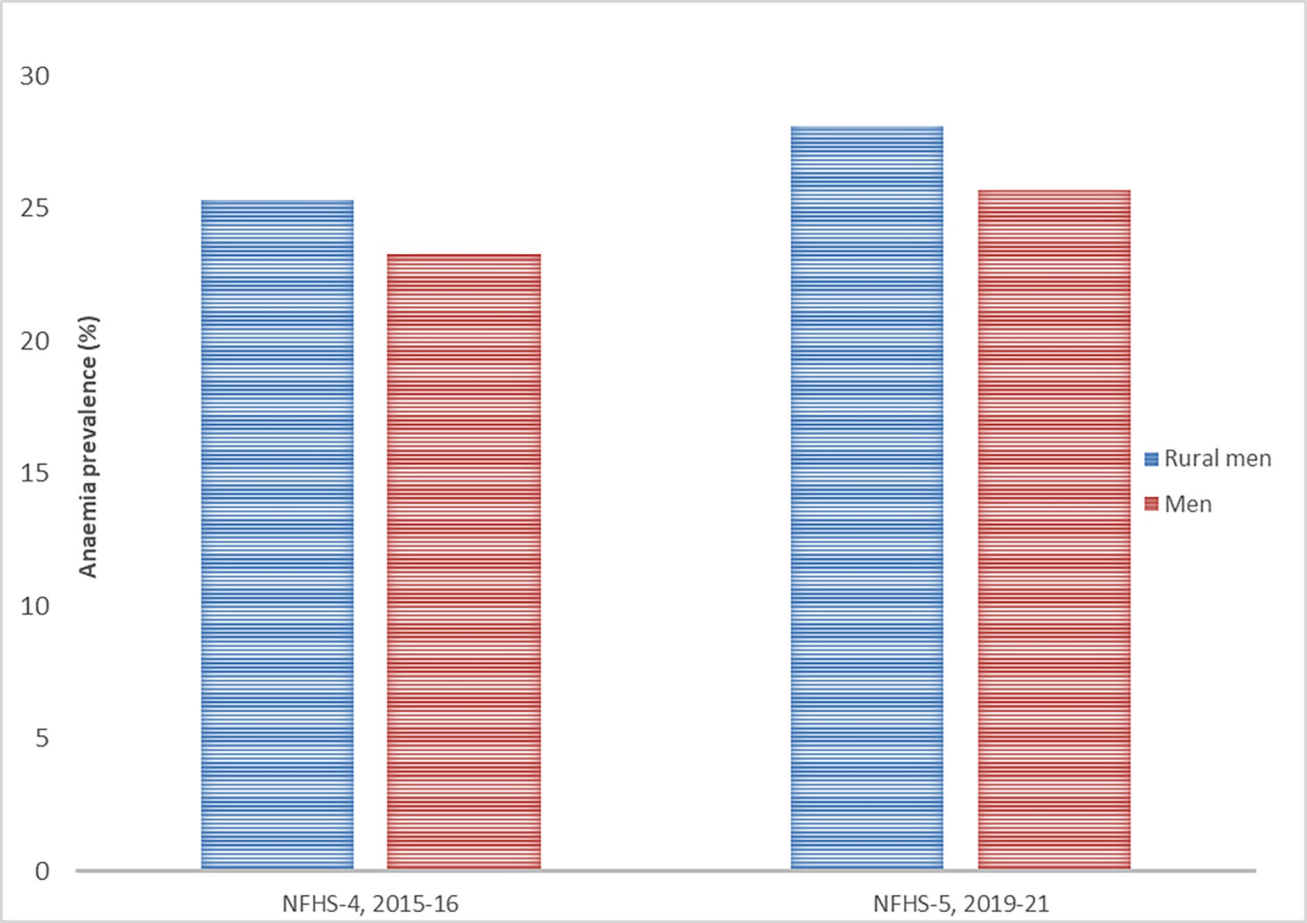
Trend of anaemia prevalence among rural men and men overall in India, NFHS.

Table 3 depicts the prevalence of anaemia among rural men in the country by various background characteristics. Overall, about one-fourth men in India were found to have anaemia. One out of every five urban men while three out of every ten rural men were anaemic in India (Fig 3). The prevalence of anaemia among rural men was highest in the age group 50–54 (34.1%) followed by the age group 15–19 years (33.8%). Men aged 20–29 years (22.9%) had the lowest prevalence of anaemia. Prevalence of anaemia decreased with increase in education. Men with no education had the highest prevalence of anaemia. ST men (30.9%) showed the highest prevalence of anaemia among the social groups. Anaemia prevalence was significantly higher among Muslim men and lower among Christian men. A steady decline was observed in the prevalence of anaemia with increase in household wealth. About one-third rural men belonging to the poorest households had anaemia.

**Table 3:** Differentials in the prevalence of anaemia among rural men by background characteristics, India, 2019–21.

Background characteristics	Prevalence of anaemia (%)	P value	Chi-Square Value
**Age (in years)**		<0.001	615.93
15–19	33.8		
20–29	22.9		
30–39	23.8		
40–49	27.0		
50–54	34.1		
**Level of education**		<0.001	404.12
No education	32.3		
Primary	29.0		
Secondary	26.5		
Higher	20.0		
**Social groups**		<0.001	230.57
SC	26.1		
ST	30.9		
OBC	24.6		
Others	24.8		
**Religion**		<0.001	227.5
Hindu	26.6		
Muslim	32.3		
Christian	20.3		
Others	27.2		
**Household wealth**		<0.001	654.25
Poorest	32.8		
Poorer	27.6		
Middle	24.7		
Richer	22.1		
Richest	19.9		
**Marital status**		<0.001	51.81
Never married	28.0		
Currently married	25.9		
Formerly in union	32.3		
**Work status**		<0.001	66
No	29.0		
Yes	25.9		
**Region**		<0.001	691.88
South	18.5		
North	27.2		
Central	25.0		
East	34.1		
West	28.9		
North-East	26.9		
**Body Mass Index**		<0.001	686.22
Underweight	34.7		
Normal	26.7		
Overweight	20.3		
Obese	19.3		
**Media exposure**		<0.001	153.54
No	29.1		
Yes	24.9		
**Consumption of non-vegetarian food**		0.129	2.29
No	26.3		
Yes	26.9		
**Alcohol consumption**		<0.001	76.01
Never	27.4		
Less than once a week	23.4		
Once a week	24.8		
daily	28.6		
**Currently smokes**		0.001	11.38
No	26.2		
Yes	27.3		
**Use of smokeless tobacco**		<0.001	32.52
No	26.1		
Yes	28.2		
**Total**	28.1		

**Notes:** P value based on chi-square test.

Anaemia prevalence was highest in the eastern region (34.1%) while lowest in the southern region (18.5%). The north, west, north-west and central regions reported 27.2%, 28.9%, 26.9% and 25% prevalence of anaemia, respectively. Anaemia was inversely related with BMI as prevalence of anaemia was 34.7% among underweight men versus 19.3% among men who were overweight. Men who drank alcohol daily and used smokeless tobacco had slightly higher occurrence of anaemia than who did not consume it.

### Estimates from logistic regression analysis for anaemia among men in rural India

The results from the logistic regression models are presented in Table 4. The men within the age bracket 20–29 years and 30–39 years were 33% and 26% less likely to be anaemic than men aged 15–19. Men aged 50–54 years were slightly more likely to be anaemic (OR: 1.10, 95% CI, 1.00–1.21). Men with no formal education were 36% more likely (95% CI, 1.26–1.47) to be anaemic than men who obtained higher education. Men with primary education were a quarter times more likely (95% CI, 1.15–1.34) to have anaemia as compared to men with higher education. Men from to ST category (OR: 1.48, 95% CI, 1.39–1.58) had significantly higher likelihood of being anaemic as compared to men of ‘Others’ category. The odds of being anaemic were 36% higher among Muslim men (95% CI, 1.27–1.45) and 48% lower among Christian men (95% CI, 0.48–0.57) as compared to Hindu men. The more the wealth, the lesser the risk to suffer from anaemia. Men from the richest wealth quintile were 29% less likely to suffer from anaemia (95% CI, 0.66–0.78) than those from the poorest wealth quintile.

**Table 4:** Odds ratios (with 95% CI) for anaemia among men by their background characteristics, India, 2019–21.

	Model 1				Model 2				Model 3				Model 4			
		CI (95%)				CI (95%)				CI (95%)				CI (95%)		
Independent Variables	OR	Upper	Lower	P value	OR	Upper	Lower	P value	OR	Upper	Lower	P value	OR	Upper	Lower	P value
***Socioeconomic variables*** **Age**																
15–19	Ref.				Ref.				Ref.				Ref.			
20–29	0.62	0.58	0.66	0.000	0.61	0.57	0.65	0.000	0.66	0.62	0.70	0.000	0.67	0.63	0.71	0.000
30–39	0.65	0.60	0.70	0.000	0.65	0.60	0.70	0.000	0.72	0.67	0.78	0.000	0.74	0.69	0.80	0.000
40–49	0.79	0.73	0.86	0.000	0.79	0.73	0.86	0.000	0.88	0.81	0.96	0.002	0.91	0.84	0.99	0.032
50–54	0.95	0.86	1.04	0.027	0.95	0.87	1.04	0.286	1.06	0.96	1.16	0.252	1.10	1.00	1.21	0.052
**Level of education**																
No education	1.36	1.26	1.46	0.000	1.38	1.28	1.18	0.000	1.32	1.13	1.43	0.000	1.36	1.26	1.47	0.000
Primary	1.26	1.17	1.36	0.000	1.25	1.16	1.35	0.000	1.21	1.12	1.30	0.000	1.24	1.15	1.34	0.000
Secondary	1.18	1.11	1.25	0.000	1.17	1.10	1.24	0.000	1.14	1.08	1.21	0.000	1.16	1.09	1.23	0.000
Higher	Ref.				Ref.				Ref.				Ref.			
**Social group**																
Others	Ref.				Ref.				Ref.				Ref.			
SC	1.03	0.97	1.09	0.345	1.08	1.01	1.15	0.016	1.06	0.99	1.12	0.072	1.07	1.00	1.13	0.044
SC	1.43	1.34	1.52	0.000	1.47	1.38	1.57	0.000	1.47	1.38	1.56	0.000	1.48	1.39	1.58	0.000
OBC	0.94	0.90	0.99	0.029	1.01	0.96	1.07	0.725	1.00	0.94	1.05	0.877	0.99	0.94	1.05	0.783
**Religion**																
Hindu	Ref.				Ref.				Ref.				Ref.			
Muslim	1.40	1.31	1.49	0.000	1.35	1.27	1.44	0.000	1.38	1.29	1.47	0.000	1.36	1.27	1.45	0.000
Christian	0.51	0.48	0.56	0.000	0.52	0.47	0.56	0.000	0.52	0.48	0.57	0.000	0.52	0.48	0.57	0.000
Others	0.98	0.91	1.06	0.618	0.91	0.84	0.99	0.025	0.94	0.86	1.02	0.109	0.94	0.87	1.02	0.139
**Household wealth**																
Poorest	Ref.				Ref.				Ref.				Ref.			
Poorer	0.85	0.81	0.89	0.000	0.88	0.84	0.92	0.000	0.90	0.86	0.94	0.000	0.89	0.85	0.94	0.000
Middle	0.76	0.72	0.80	0.000	0.81	0.77	0.86	0.000	0.85	0.80	0.90	0.000	0.84	0.80	0.89	0.000
Richer	0.68	0.64	0.72	0.000	0.73	0.69	0.78	0.000	0.78	0.73	0.83	0.000	0.77	0.72	0.82	0.000
Richest	0.63	0.58	0.68	0.000	0.66	0.61	0.72	0.000	0.72	0.66	0.79	0.000	0.71	0.66	0.78	0.000
**Work status**																
No	Ref.															
Yes	0.94	0.90	0.99	0.013												
**Marital status**																
Never in union	Ref.				Ref.				Ref.				Ref.			
Currently in union	0.96	0.90	1.02	0.162	0.93	0.88	0.98	0.013	0.96	0.90	1.02	0.162	0.97	0.91	1.03	0.286
Formerly in union	1.20	1.04	1.38	0.012	1.18	1.02	1.36	0.023	1.20	1.04	1.38	0.014	1.22	1.05	1.40	0.007
***Geographic variable*** **Region**																
Central					Ref.				Ref.				Ref.			
South					0.78	0.73	0.84	0.000	0.80	0.75	0.86	0.000	0.81	0.76	0.87	0.000
North					1.25	1.18	1.32	0.000	1.27	1.20	1.35	0.000	1.27	1.20	1.35	0.000
East					1.44	1.36	1.52	0.000	1.45	1.37	1.53	0.000	1.47	1.39	1.55	0.000
West					1.32	1.24	1.40	0.000	1.30	1.22	1.39	0.000	1.28	1.20	1.36	0.000
Northeast					1.16	1.09	1.24	0.000	1.21	1.13	1.29	0.000	1.24	1.16	1.33	0.000
***Health-related variables*** **Body Mass Index**																
Normal									Ref.				Ref.			
Underweight									1.36	1.30	1.43	0.000	1.36	1.30	1.43	0.000
Overweight									0.76	0.72	0.80	0.000	0.76	0.72	0.80	0.000
Obese									0.76	0.68	0.86	0.000	0.77	0.68	0.86	0.000
**Blood sugar**																
Normal									Ref.							
High									1.07	0.99	1.15	0.086				
Very High									1.07	0.98	1.17	0.141				
***Behavioural variables*** **Media exposure**																
No													Ref.			
Yes													1.03	1.00	1.08	0.087
**Consumption of non-vegetarian food**																
No													Ref.			
Yes													1.03	0.98	1.07	0.217
**Alcohol consumption**																
Never													Ref.			
Less than once a week													0.85	0.80	0.90	0.000
Once a week													0.91	0.85	0.96	0.001
Almost every day													0.96	0.88	1.05	0.412
**Currently smokes**																
No													Ref.			
Yes													1.15	1.08	1.22	0.022
**Use of smokeless tobacco**																
No													Ref.			
Yes													1.07	1.01	1.13	0.016

**Notes:** OR: Odds ratio, CI: Confidence interval, Ref.: Reference category.

The odds of anaemia among men from the east region of the country were 47% (95% CI, 1.39–1.55) higher than those from the central region. Men belonging to the north, west and north-east regions were 27%, 28%, and 24% more likely to suffer from anaemia. However, the odds of anaemia among men were lower by 19% (95% CI, 0.76–0.87) in the south region. Men who were underweight had 36% (95% CI, 1.30–1.43) more likelihood of being anaemic whereas obese men were 23% (95% CI, 0.68–0.86) less likely to suffer from anaemia as compared to men with normal BMI. The risk of anaemia among men using smokeless tobacco was more (OR:1.15, 95% CI, 1.08–1.22) than those not using the same.

### Spatial analysis

**Fig 4:**
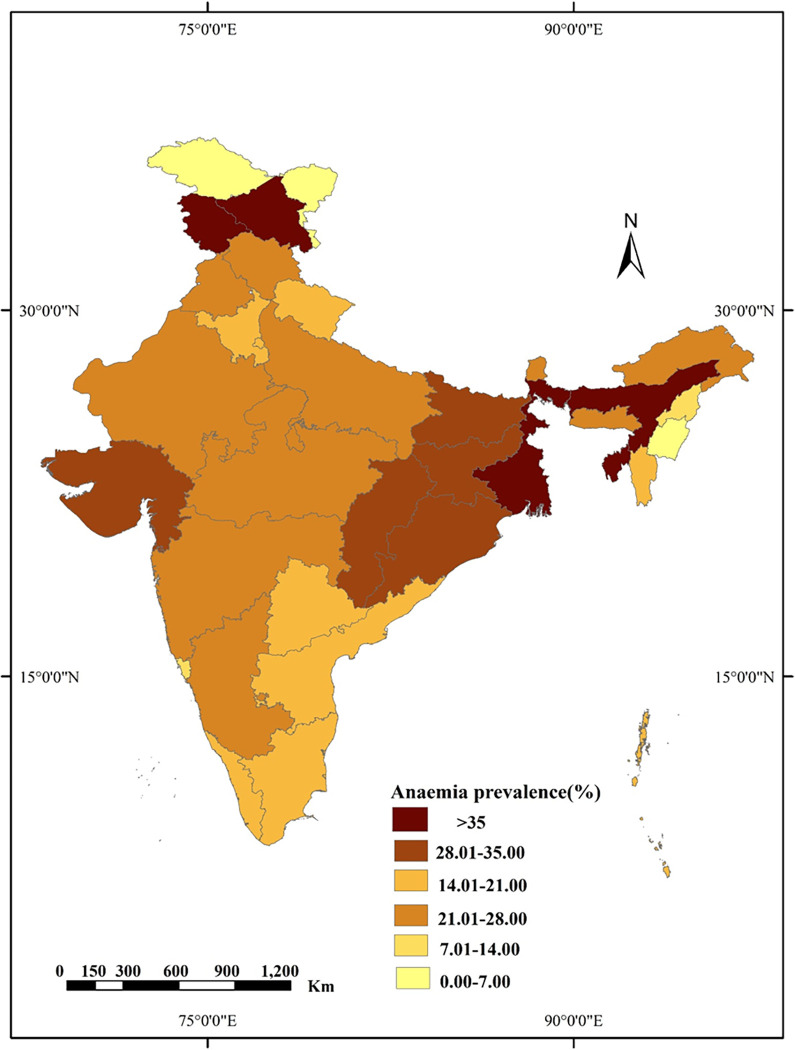
Map showing state wise prevalence anaemia among rural men in India, NFHS-5, 2019–21. (All the maps used are the authors’ own creations. As far as the base layer is concerned, we used a free GIS file from https://spatialdata.dhsprogram.com/boundaries/#view=table&countryId=IA for national and sub-national boundaries).

**Fig 5:**
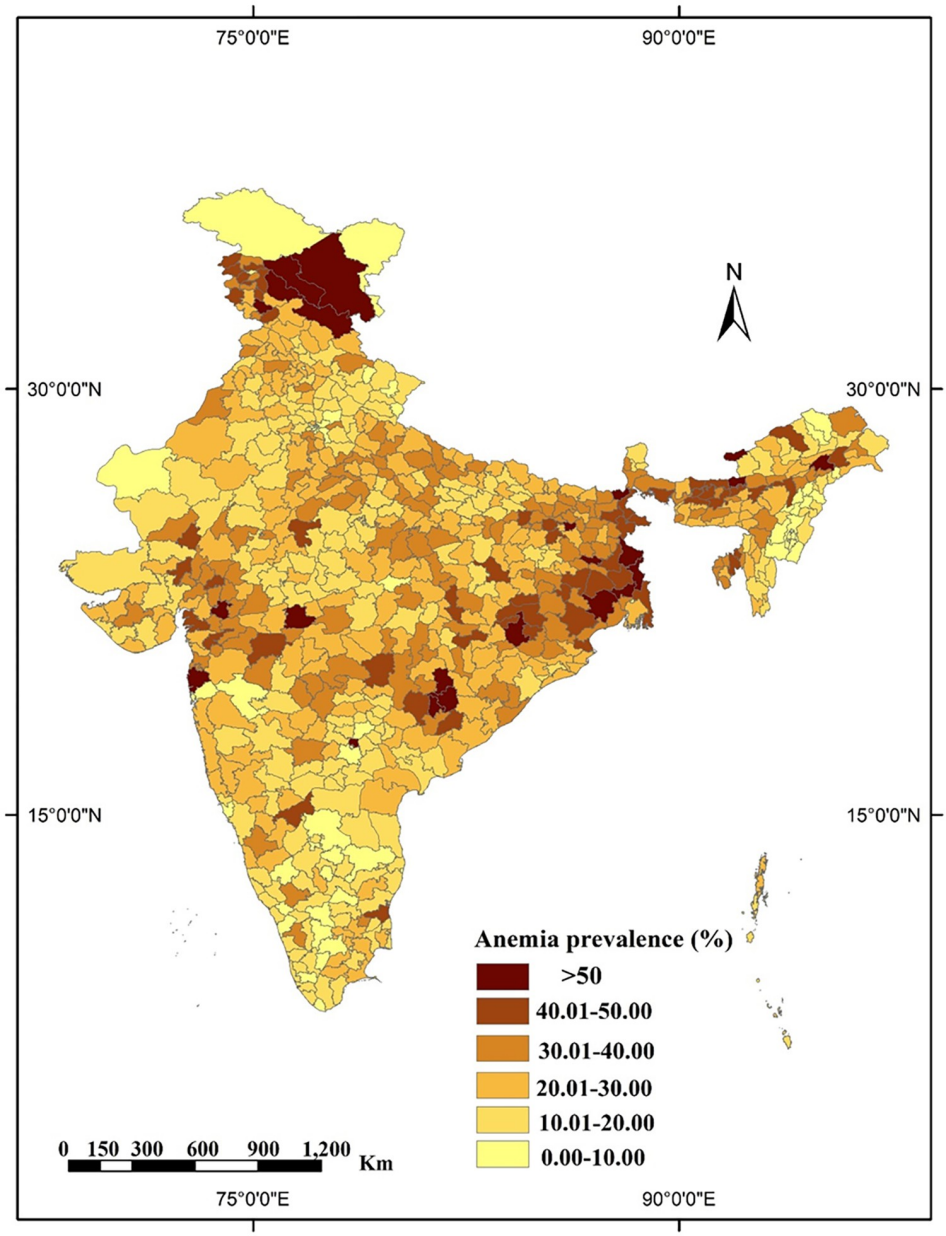
Map showing district wise prevalence of anaemia among rural men in India, NFHS-5, 2019–21. (All the maps used are the authors’ own creations. As far as the base layer is concerned, we used a free GIS file from https://spatialdata.dhsprogram.com/boundaries/#view=table&countryId=IA for national and sub-national boundaries).

Figs 4 and 5 show the spatial distribution of prevalence of anaemia among men across the Indian states and districts respectively using choropleth map. It is a technique through which unequal distribution of an element within a geographic area is depicted through gradients of the same colour. The higher the value or prevalence, the darker the shade [31].

The states as well as districts of India were classified into six categories according to prevalence of anaemia (%). West Bengal, Tripura, Assam, UT Jammu and Kashmir (>35%) were found to have highest anaemia prevalence among rural men followed by Bihar, Jharkhand, Chhattisgarh, Odisha, Gujarat (28%-35%). South Indian states i.e., Andhra Pradesh, Karnataka, Tamil Nadu, and Kerala,) as well as Manipur and Nagaland showed lowest prevalence.

Districts of West Bengal, Bihar, Odisha, Chhattisgarh, Jammu & Kashmir (>50%) showed the highest prevalence of anaemia among rural men followed by some districts of Uttar Pradesh and Madhya Pradesh (40%-50%). The lowest prevalence was found in some districts of Manipur, Nagaland, Karnataka, and Tamil Nadu (<10%).

## Discussion

An estimated one-fourth of Indian rural men aged 15 to 54 years were found to be anaemic. In the last four years, the prevalence of anaemia has only risen [8]. The findings suggested that the prevalence of anaemia varies by sociodemographic characteristics among rural men. Rural men who were 49–54 years old, had no formal education, belonged to ST group, were Muslim, or from the poorest wealth quintile, lived in the eastern region, were underweight, and consumed alcohol and smokeless tobacco on a daily basis were more likely to have anaemia. At both the state and district levels, there was significant geographical variation in the prevalence of anaemia.

Older men, aged 50–54 years, were more likely to be anaemic, followed by adolescents (15–19 years). However, the prevalence of anaemia was lowest in the 20–29 age group. Older men are more vulnerable to anaemia, possibly as a result of suffering from other chronic diseases such as diabetes, chronic kidney diseases, hypertension [32]. Previous research has yielded similar results [26,33], although none of these studies have been conducted specifically on rural men.

Education level emerged to be a significant determinant of anaemia. Rural men with no education were most vulnerable to anaemia. Men with a higher level of education were less likely to develop anaemia. Previous studies also corroborate the same [19,34]. Disease awareness and knowledge, as well as the necessity of sanitation and health care, are raised through education. It also encourages people to listen to and accept the advice of health professionals [35,36].

Men in the ST category were more likely to be anaemic than men from other social groups. STs have a long history of being marginalised, with the majority of them still living in remote areas of the country. As a result, poor diet and a lack of access to healthcare services could be linked to their increased risk of anaemia [37]. Various studies have found that SCs and STs generally have poor health outcomes, underscoring the importance of caste prejudices. Despite affirmative actions by the Indian government post-1947, people from SC/ST groups remain deprived in a variety of areas, including health [38–40].

Muslim men had greater risk of anaemia, which is similar to the findings from an earlier study [20]. This study also found that Christian men living in rural India were at significantly lower risk of anaemia, which was not highlighted previously by any scientific study and requires further investigation. However, these results need to be cautiously interpreted as the sample size for Muslim and Christian population was small and sample distribution was highly skewed.

Household wealth was strongly associated with anaemia. The wealthier the household, the lesser the risk of anaemia. Several researches have explored the linkage between poverty and malnutrition. Poverty makes it difficult for people to eat a healthy diet and get health care [41,42]. Low socioeconomic position can exacerbate the prevalence of anaemia in a variety of ways, including a poor living and working environment, unhealthy habits such as smoking and limited access to health care, and a lack of health literacy [40]. In developing countries, people from poorer households are more likely to suffer anaemia than those from wealthy homes [43] due to factors such as substandard housing, hunger, and increased disease exposure.

A significant geographical variation in the prevalence of anaemia among rural men was also noted in this study. The likelihood of being anaemic was maximum among men belonging to the eastern region. A recent study on anaemia among men also pointed out higher prevalence of anaemia among men in the eastern India [21]. A previous study on anaemia among children also highlighted that people from central and eastern region were associated with higher risk of being anaemic [44]. However, this study found that men from the north, west and north-east region were more likely to suffer from anaemia compared to men in the central region. The differentials in population composition from one region to another could play a vital role in spatial variation in anaemia.

Anaemia was significantly associated with BMI in this study. Rural men with lower BMI, who were underweight, had a higher risk of anaemia. It is a well-researched fact that underweight persons have higher likelihood to be anaemic, as low BMI is caused by a lack of a balanced and healthy diet [45]. Men who drank alcohol on a regular basis had a higher risk of anaemia than men who did not consume alcohol. A population based study on India offered a similar finding [19]. Frequent consumption of alcohol leads to deterioration of health and a number of other chronic illnesses which are linked with anaemia.

There was no significant relationship found between anaemia prevalence and media exposure, blood sugar, and non-vegetarian food consumption. Previous studies have found a link between anaemia and these variables. It has been noted that as blood sugar levels rise, the likelihood of being anaemic also rises [46]. Another study on women in Afghanistan found a strong negative correlation between anaemia risk and meat consumption frequency [47]. Since the above-mentioned study was conducted on women, we should exercise caution while comparing the results of the current study with the findings from other studies. It was also found that women who took iron tablets or syrup at regular intervals had a lower risk of anaemia [48]. Thus, further research is needed to investigate the effect of such interventions in men.

The Indian government has devised a number of programmes and policies aimed at reducing the prevalence of anaemia in the country. Almost all of them, however, primarily target women of reproductive age and children. The National Nutritional Anaemia Control Program (NNACP), for example, was established in 1970 with the goals of encouraging regular consumption of iron-rich foods, providing iron and folate supplements to susceptible groups, and identifying and treating severely anaemic patients [49]. The 12-by-12 Initiative (2007) was launched in collaboration with the Ministry of Health and Family Welfare, the World Health Organization (WHO), UNICEF, and the Food and Agriculture Organization of the United Nations, with the goal of every child having a haemoglobin level of 12 grams by the age of 12 years by 2012 [50]. The National Iron Plus Initiative (NIPI) was launched in 2013 with the goal of providing free iron and folic acid supplements to adolescent boys and girls (10–19 years) as well as women of reproductive age, such as pregnant and breastfeeding women. The Union Ministry of Health and Family Welfare started the Weekly Iron and Folic Acid Supplementation (WIFS) initiative in 2013 under the National Health Mission to prevent anaemia among teenagers (NHM). Another initiative, Anaemia Mukt Bharat (2018), aimed to cut anaemia in young children, teenage boys and girls, pregnant and breastfeeding women, and women of reproductive age by half [50]. It is therefore recommended that the government and policymakers expand the scope of programs such as Anaemia Mukt Bharat and Iron supplementation programs to include sub-strata of rural aged men who are more susceptible to anaemia. Additionally, more research is needed to design such health interventions for men, keeping the sociodemographic and cultural context in sight.

This study has a few limitations that should be mentioned. First, because the data for this study comes from a cross-sectional survey, the relationship between dependent and independent variables demonstrated in this paper should be interpreted as association, and not as causality. Second, the model in this study uses only those variables that were available in the dataset. Some predictor variables may have been left out which may have resulted in what is known as omitted variable bias. We were unable to include folate, vitamin B12, or vitamin A intake as predictor variables in the model due to a lack of data. Third, the nationwide representative survey measured haemoglobin concentrations with a battery-operated portable HemoCueHb 201+ analyser, which may have underestimated the results when compared to laboratory testing (Didzun et al., 2019). Future research should take these limitations into account to get a more accurate and comprehensive picture of the prevalence of anaemia among rural men in India.

## Conclusion

Anaemia among rural men, just like it is among women and children, is a serious public health concern in India. Anaemia was found in three out of ten rural men. High-risk groups were older men, men without education, Muslim and STs, men from the poorest households, and men who were underweight. The benefits of existing programs and policies related to anaemia eradiation should be extended to men as well. In addition, targeted interventions among susceptible groups of rural men are advised as a way to reduce the prevalence of anaemia. Men’s haemoglobin levels should be checked on a regular basis and for that purpose appropriate screening facilities should to be made available closer to their residences so that they can be screened easily. When developing policies, it is important to keep geographical regions with high anaemia prevalence in mind. A comprehensive strategy based on the aforementioned proposals could be beneficial to reduce burden of anaemia among men in rural India.

## References

[1] WHO. Health Topics. Anaemia. World Health Organisation. 2022.

[2] Marengo-rowe AJ. Bumc0019-0239. Proc Baylor Univ Med Cent. 2006;19: 239–245. 10.1080/08998280.2006.11928171 PMC1484532 17252042

[3] WHO. Nutritional Anaemias: Tools for Effective Prevention. World Health Organization. 2017.

[4] Kassebaum NJ. The Global Burden of Anemia GBD 2013 Anemia Collaborators and Nicholas J Kassebaum. Hematol Clin. 2016;30: 247–308. 10.1016/j.hoc.2015.11.002 27040955

[5] WHO. Global nutrition targets 2025: Anaemia policy brief (WHO/NMH/NHD/14.4). Geneva: World Health Organization; 2014. Geneva; 2014.

[6] Goal 2 | Department of Economic and Social Affairs.

[7] WHO. Global Nutrition Report: 2021. Glob Nutr Rep. 2021.

[8] International Institute for Population Sciences (IIPS) and ICF. 2021. National Family Health Survey (NFHS-5), 2019–21: India. Mumbai; 2021.

[9] Haider BA, Olofin I, Wang M, Spiegelman D, Ezzati M, Fawzi WW. Anaemia, prenatal iron use, and risk of adverse pregnancy outcomes: Systematic review and meta-analysis. BMJ. 2013;347: 1–19. doi: 10.1136/bmj.f3443 23794316 PMC3689887

[10] Mireku MO, Davidson LL, Koura GK, Ouédraogo S, Boivin MJ, Xiong X, et al. Prenatal hemoglobin levels and early cognitive and motor functions of one-year-old children. Pediatrics. 2015;136: e76–e83. doi: 10.1542/peds.2015-0491 26055847 PMC9924076

[11] Scott SP, Chen-Edinboro LP, Caulfield LE, Murray-Kolb LE. The impact of anemia on child mortality: An updated review. Nutrients. 2014;6: 5915–5932. doi: 10.3390/nu6125915 25533005 PMC4277007

[12] Daru J, Zamora J, Fernández-Félix BM, Vogel J, Oladapo OT, Morisaki N, et al. Risk of maternal mortality in women with severe anaemia during pregnancy and post partum: a multilevel analysis. Lancet Glob Heal. 2018;6: e548–e554. doi: 10.1016/S2214-109X(18)30078-0 29571592

[13] Horton S, Ross J. Corrigendum to: ‘“The Economics of iron deficiency”‘ [Food Policy 28 (2003) 51–75]. 2007;32: 141–143. doi: 10.1016/j.foodpol.2006.08.002

[14] J.M. H. Reversing productivity losses from iron deficiency: The economic case. J Nutr. 2002;132: 794S–801S. doi: 10.1093/jn/132.4.794S 11925484

[15] Indian Council of Medical Research, Public Health Foundaton of India I for HM and E. India: Health of the Nation ‘ s States- The India State-Level Disease Burden Initiative. New Delhi, India. New Delhi, India.; 2017.

[16] Islam GMR. Inequality, chronic undernutrition, maternity, and diabetes mellitus as the determinant of anemia among ever-married women in Bangladesh. BMC Public Health. 2021;21: 1–11. doi: 10.1186/s12889-021-10362-2 33549086 PMC7866870

[17] Stevens GA, Finucane MM, De-Regil LM, Paciorek CJ, Flaxman SR, Branca F, et al. Global, regional, and national trends in haemoglobin concentration and prevalence of total and severe anaemia in children and pregnant and non-pregnant women for 1995–2011: A systematic analysis of population-representative data. Lancet Glob Heal. 2013;1: 16–25. doi: 10.1016/S2214-109X(13)70001-9 25103581 PMC4547326

[18] Nguyen PH, Scott S, Avula R, Tran LM, Menon P. Trends and drivers of change in the prevalence of anaemia among 1 million women and children in India, 2006 to 2016. BMJ Glob Heal. 2018;3: 1–12. doi: 10.1136/bmjgh-2018-001010 30397516 PMC6202996

[19] Didzun O, De Neve JW, Awasthi A, Dubey M, Theilmann M, Bärnighausen T, et al. Anaemia among men in India: a nationally representative cross-sectional study. Lancet Glob Heal. 2019;7: e1685–e1694. doi: 10.1016/S2214-109X(19)30440-1 31708149

[20] Kumar P, Sharma H, Sinha D. Socio-economic inequality in anaemia among men in India: a study based on cross-sectional data. BMC Public Health. 2021;21: 1–12. doi: 10.1186/s12889-021-11393-5 34233633 PMC8265140

[21] Kumar P, Sharma H, Patel KK. Prevalence and risk factors of anaemia among men: A study based on Empowered Action Group states, India. Nutr Health. 2021;27: 191–198. doi: 10.1177/0260106020982348 33472523

[22] Cohen AR, Seidl-Friedman J. HemoCue system for hemoglobin measurement. Evaluation in anemic and nonanemic children. Am J Clin Pathol. 1988;90: 302–305. doi: 10.1093/ajcp/90.3.302 3414603

[23] Vanzetti G. An azide-methemoglobin method for hemoglobin determination in blood. J Lab Clin Med. 1966;67: 116–126. 5900720

[24] Sanchis-Gomar F, Cortell-Ballester J, Pareja-Galeano H, Banfi G, Lippi G. Hemoglobin Point-of-Care Testing: The HemoCue System. J Lab Autom. 2013;18: 198–205. doi: 10.1177/2211068212457560 22961038

[25] Khusun H, Ray Y, Schultink W, Dillon DHS. World health organization hemoglobin cut-off points for the detection of anemia are valid for an Indonesian population. J Nutr. 1999;129: 1669–1674. doi: 10.1093/jn/129.9.1669 10460202

[26] Awaluddin SM, Shahein NA, Rahim NCA, Zaki NAM, Nasaruddin NH, Saminathan TA, et al. Anemia among men in Malaysia: A population-based survey in 2019. Int J Environ Res Public Health. 2021;18. doi: 10.3390/ijerph182010922 34682667 PMC8535807

[27] Kant S, Kumar R, Malhotra S, Kaur R, Haldar P. Prevalence and determinants of anemia among adult males in a rural area of Haryana, India. J Epidemiol Glob Health. 2019;9: 128–134. doi: 10.2991/jegh.k.190513.001 31241871 PMC7310746

[28] Singh RK, Patra S. Extent of Anaemia among Preschool Children in EAG States, India: A Challenge to Policy Makers. Anemia. 2014;2014. doi: 10.1155/2014/868752 25140250 PMC4129919

[29] StataCorp. Stata: Statistical Software. College Station, TX: College Station, TX: StataCorp LLC.; 2019.

[30] ESRI. ArcMap Software. Redlands, CA: ESRI INC, 2016.; 2016.

[31] Chien TW, Wang HY, Hsu CF, Kuo SC, Liu M. Choropleth map legend design for visualizing the most influential areas in article citation disparities: A bibliometric study. Med (United States). 2019;98. doi: 10.1097/MD.0000000000017527 31593127 PMC6799475

[32] Paul B, Wilfred NC, Woodman R, DePasquale C. Prevalence and correlates of anaemia in essential hypertension. Clin Exp Pharmacol Physiol. 2008;35: 1461–1464. doi: 10.1111/j.1440-1681.2008.05031.x 18759858

[33] Duman TT, Aktas G, Meryem Atak B, Kocak MZ, Kurtkulagi O, Bilgin S. General characteristics of anemia in postmenopausal women and elderly men. Aging Male. 2021;23: 780–784. doi: 10.1080/13685538.2019.1595571 30945964

[34] Adamu AL, Crampin A, Kayuni N, Amberbir A, Koole O, Phiri A, et al. Prevalence and risk factors for anemia severity and type in Malawian men and women: Urban and rural differences. Popul Health Metr. 2017;15: 1–15. doi: 10.1186/s12963-017-0128-2 28356159 PMC5371260

[35] Sunuwar DR, Sangroula RK, Shakya NS, Yadav R, Chaudhary NK, Pradhan PMS. Effect of nutrition education on hemoglobin level in pregnant women: A quasi-experimental study. PLoS One. 2019;14: 1–12. doi: 10.1371/journal.pone.0213982 30897129 PMC6428266

[36] Raghupathi V, Raghupathi W. The influence of education on health: An empirical assessment of OECD countries for the period 1995–2015. Arch Public Heal. 2020;78: 1–18. doi: 10.1186/s13690-020-00402-5 32280462 PMC7133023

[37] Singh A, Kumar A, Kumar A. Determinants of neonatal mortality in rural India, 2007–2008. 2013; 2007–2008. doi: 10.7717/peerj.75 23734339 PMC3669267

[38] Van De Poel E, Speybroeck N. Decomposing malnutrition inequalities between Scheduled Castes and Tribes and the remaining Indian population. Ethn Heal. 2009;14: 271–287. doi: 10.1080/13557850802609931 19259879

[39] Dommaraju P, Agadjanian V, Yabiku S. The pervasive and persistent influence of caste on child mortality in India. Popul Res Policy Rev. 2008;27: 477–495. doi: 10.1007/s11113-008-9070-0

[40] Vart P, Jaglan A, Shafique K. Caste-based social inequalities and childhood anemia in India: Results from the National Family Health Survey (NFHS) 2005–2006 Chronic Disease epidemiology. BMC Public Health. 2015;15: 1–8. doi: 10.1186/s12889-015-1881-4 26044618 PMC4456806

[41] Kibret KT, Chojenta C, D’Arcy E, Loxton D. Spatial distribution and determinant factors of anaemia among women of reproductive age in Ethiopia: A multilevel and spatial analysis. BMJ Open. 2019;9. doi: 10.1136/bmjopen-2018-027276 30948614 PMC6500301

[42] Sunuwar DR, Singh DR, Adhikari B, Shrestha S, Pradhan PMS. Factors affecting anaemia among women of reproductive age in Nepal: A multilevel and spatial analysis. BMJ Open. 2021;11. doi: 10.1136/bmjopen-2020-041982 33782019 PMC8009228

[43] Balarajan Y, Ramakrishnan U, Özaltin E, Shankar AH, Subramanian S V. Anaemia in low-income and middle-income countries. Lancet. 2011;378: 2123–2135. doi: 10.1016/S0140-6736(10)62304-5 21813172

[44] Sharma H, Singh SK, Srivastava S. Socio-economic inequality and spatial heterogeneity in anaemia among children in India: Evidence from NFHS-4 (2015–16). Clin Epidemiol Glob Heal. 2020;8: 1158–1171. doi: 10.1016/j.cegh.2020.04.009

[45] Pal A, De S, Sengupta P, Maity P, Dhara PC. An investigation on prevalence of Anaemia in relation to BMI and nutrient intake among adult rural population of West Bengal, India. Epidemiol Biostat Public Heal. 2014;11: 1–10. doi: 10.2427/8915

[46] Soliman AT, De Sanctis V, Yassin M, Soliman N. Iron deficiency anemia and glucose metabolism. Acta Biomed. 2017;88: 112–118. doi: 10.23750/abm.v88i1.6049 28467345 PMC6166192

[47] Flores-Martinez A, Zanello G, Shankar B, Poole N. Reducing anemia prevalence in Afghanistan: Socioeconomic correlates and the particular role of agricultural assets. PLoS One. 2016;11: 1–23. doi: 10.1371/journal.pone.0156878 27271735 PMC4894627

[48] Wendt A, Stephenson R, Young M, Webb-Girard A, Hogue C, Ramakrishnan U, et al. Individual and Facility-Level Determinants of Iron and Folic Acid Receipt and Adequate Consumption among Pregnant Women in Rural Bihar, India. PLoS One. 2015;10: e0120404. doi: 10.1371/journal.pone.0120404 25793866 PMC4368810

[49] Kumar A. National nutritional anaemia control programme in India. Indian J Public Health. 1999;43: 3–5,16. 11243085

[50] Bhatia PV, Sahoo DP, Parida SP. India steps ahead to curb anemia: Anemia Mukt Bharat. Indian J Community Heal. 2018;30: 312–316.

